# Detection of G12 Human Rotaviruses in Nepal

**DOI:** 10.3201/eid1303.061367

**Published:** 2007-03

**Authors:** Sher Bahadur Pun, Toyoko Nakagomi, Jeevan Bahadur Sherchand, Basu Dev Pandey, Luis E. Cuevas, Nigel A. Cunliffe, C.A. Hart, Osamu Nakagomi

**Affiliations:** * Nagasaki University, Nagasaki, Japan; † University of Liverpool, Liverpool, United Kingdom; ‡ Tribhuvan University Institute of Medicine, Kathmandu, Nepal; § Infectious and Tropical Diseases Research Centre, Kathmandu, Nepal; ¶ Sukra Raj Tropical and Infectious Disease Hospital, Kathmandu, Nepal; # Liverpool School of Tropical Medicine, Liverpool, United Kingdom

**Keywords:** rotavirus, serotype, genotyping, PCR, G12, molecular epidemiology, dispatch

## Abstract

Of 731 stool specimens collected from children with diarrhea in Kathmandu, Nepal, from August 2004 through July 2005, 170 (23.3%) tested positive for rotavirus. Reverse transcription–PCR, including a revised G12-specific primer set, identified 56 (32.9%) as G2P[4] and 39 (23.0%) as G12 with P[6], P[8], or P[4].

Globally, ≈700,000 children each year die of rotavirus diarrheal disease, and most such deaths occur in developing countries (*1*,*2*). To reduce this substantial burden of rotavirus diarrhea, 2 live, attenuated oral vaccines have been licensed in >60 countries and are being introduced into the areas where they are most needed (*3*). Nepal is a small, poor, landlocked, subtropical country in Asia where the severe diarrhea is common. During 2003–2004, before Nepal was formally integrated into the Asian Rotavirus Surveillance Network (*4*), a surveillance study was initiated, which showed the emergence of G12 strains against a background of predominant G1P[8] strains (*5*). Since the prevalence of G12 strains was unusually high (20%), and such strains may not be adequately covered by existing vaccines, there was an urgent need to continue surveillance and characterization of rotavirus strains by developing a G12-specific primer pair and to continue to monitor the prevalence of G12 strains in this population.

## The Study

Stool specimens were collected from children with acute diarrhea attending the rehydration clinic at Kanti Children’s Hospital, Kathmandu, Nepal, from August 2004 through July 2005. Rotavirus diarrhea was identified by a commercially available ELISA (Rotaclone, Meridian Bioscience, Inc., Cincinnati, OH, USA). From the rotavirus-positive specimens, genomic RNA was extracted by using a QIAamp Viral RNA Mini kit (QIAGEN Sciences, Germantown, MD, USA), and the purified RNA preparations were used to determine G and P types by reverse transcription–PCR (RT-PCR) as described by Uchida et al (*5*) and by Gouvea et al. (*6*). For the detection of G12 strains, a new primer pair was designed based on the nucleotide sequence of the VP7 gene of strain Arg720 (*7*). The forward primer, G12F, was 19 mer, corresponding to nucleotide positions 169–187 (5′-GTT GTT GTC ATG CTG CCA T-3′), and the reverse primer, G12R, was 20 mer whose sequence was complementary to nucleotide positions 471–490 (5′-A GTA CAG TAC CAA ATT TCA T-3′). A G12 strain in the stool specimen was identified by the presence of a 322-bp band after electroporesis on agarose gel after PCR typing was done on the first-round RT-PCR product under the same thermal cycling conditions, including an annealing temperature of 42°C. Sequencing was performed on the VP7 gene amplification products with primers Beg9 and End9 (Genomic Research Center, Shimadzu Corporation, Kyoto, Japan).

Of 731 stool specimens tested by Rotaclone, 170 (23.3%) were positive for rotavirus antigen. When the distribution of rotavirus-positive cases was examined, 64% occurred in infants ages 3–23 months, whereas only 2.9% occurred in infants <3 months of age. Although samples were not collected in March and April 2005, because of political instability, rotavirus likely circulated among the children in Kathmandu throughout the year. However, a seasonal variation was found in the occurrence of rotavirus diarrhea with a clear peak in January; the rotavirus detection rates varied monthly from 7.3% to 58.6%.

Of 170 rotavirus-positive specimens that underwent molecular genotyping, 4 G and 3 P genotypes were found; G2 and G12 together accounted for 62% and P[4] and P[6] together accounted for 74% of their respective genotypes (Table). A total of 10 different G and P genotype combinations were detected, which together accounted for 76% of rotavirus-positive specimens. Ten (6%) mixed infections, as defined by the specimens containing more than 1 G or P genotype, were found, and 31 (18%) specimens remained untypeable. The most common combinations were G2P[4] (33%) and G12P[6] (17%). No G4 strains were detected, nor were any P[9] strains found (which are often associated with G3 or G12 VP7 specificity). Since neither G3 nor G9 strains occurred in the preceding season in Kathmandu (*5*), their sudden appearance in Nepal needs continued attention.

**Table:** Relative frequencies of various combinations of G and P types of human rotaviruses, Kathmandu, Nepal, August 2004 through July 2005

Human rotavirus types	No. isolates (%)						Total, n (%)
	P[4]	P[6]	P[8]	P[9]	Pmix	PNT	
G1	0	5 (2.9)	9 (5.3)	0	2 (1.2)	2 (1.2)	18 (10.6)
G2	56 (32.9)	5 (2.9)	1 (0.6)	0	1 (0.6)	4 (2.3)	67 (39.4)
G3	0	10 (5.9)	0	0	0	0	10 (5.9)
G4	0	0	0	0	0	0	0
G8	0	0	0	0	0	0	0
G9	5 (2.9)	0	0	0	0	1 (0.6)	6 (3.5)
G12	2 (1.2)	29 (17.1)	7 (4.1)	0	0	1 (0.6)	39 (23)
Gmix	1 (0.6)	6 (3.5)	0	0	0	0	7 (4.1)
GNT	6 (3.5)	1 (0.6)	0	0	0	16 (9.4)	23 (13.5)
Total	70 (41.2)	56 (33)	17 (10)	0	3 (1.7)	24 (14.1)	170 (100)

**Figure 1:**
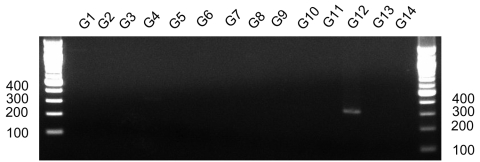
Detection after electrophoresis on a 2% agarose gel of the PCR amplification product with a primer pair G12F and G12R. The prototype rotavirus strain for each G serotype was as follows; G1, Wa; G2, KUN; G3, MO; G4, ST3; G5 OSU; G6, NCDV; G7, PO-13; G8, MW33; G9, 95H115; G10, B223; G11, YM; G12, L26; G13, L338; and G14, FI23. The first and last lanes show molecular mass markers in basepairs.

The G12 primer pair developed in this study successfully amplified 2 established G12 strains, L26 (*8*) and Se585 (*9*), and did not amplify any known G1–G11 and G13–14 strains (Figure 1). Of 39 stool specimens that were found to contain G12 rotavirus by the typing reaction with our G12 primer pair, we selected 12 specimens from those samples that produced the full-length VP7 amplification product for nucleotide sequencing. Each of these VP7 sequences was identified as G12 because the predicted amino-acid sequence was 97%–100% identical with that of prototype G12 strain Se585 in any of antigenic regions A, B, and C (*10*). GenBank accession nos. for G12 VP7 genes were AB275291 (05N054), AB275292 (05K021), AB275293 (05N128), AB275294 (04N605), AB275295 (05N140), AB275296 (05N040), AB275297 (05N065), AB275298 (05K101), AB275299 (05N138), AB275300 (05K046), AB275301 (05K066), and AB275302 (05N145).

## Conclusions

G12 strains, including those in the first human cases of infection in the Philippines in 1990 (*8*), had only been detected in sporadic cases, yet from geographically diverse locations, including the United States (*9*), Japan (*11*), Brazil (*12*), South Korea (*13*), and Thailand (*14*). However, recent investigations found that that 17% of rotaviruses collected from children with acute diarrhea during 2003–2005 in eastern India and 7.9% of those collected during 1999–2002 in Argentina were of serotype G12 (*7*,*15*). These findings were followed by our previous study, which showed that 20% of rotaviruses collected during 2003–2004 in Nepal were serotype G12 and were equally distributed between children <15 years of age and adults (*5*). The present study (2004–2005) confirms and extends these observations. Twenty-three percent of rotaviruses from the same region in the period immediately following the 2003–2004 study were serotype G12, and most (76%) were combined with P[6] and others with either P[8] or P[4], even though the dominant strains changed from G1P[8] during 2003–2004 (71%) to G2P[4] during 2004–2005 (33%). The detection of G1P[8] decreased to a minimum of 5.3% of all rotavirus-positive strains. The increasing trend of G12 strains in Nepal appears stable and may reflect the overall trend in the Ganges region at large, which justifies further surveillance in Nepal. G12 strains have similarly been detected in multiple seasons elsewhere. In Brazil, G12 strains continued to appear during 1999–2002 (*7*). The emergence and increase in G12P[6] strains may become a challenge to the current rotavirus vaccination strategy, the efficacy of which may depend on the shared G and P serotype specificity of the vaccine strains and wild-type rotavirus strains circulating among children. In this context, the detection of a G12 porcine rotavirus strain with a porcine genetic background in Kolkata, India (*16*), may indicate reservoirs of an unusual yet emerging G genotype in animals.

**Figure 2:**
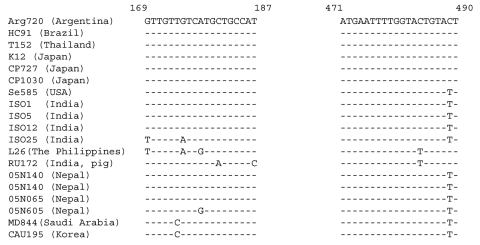
Comparison of the primer binding regions of the VP7 genes of G12 rotavirus strains detected in various geographic locations. Primers were designed based on the Arg720 sequence. The sequence of the forward primer is as shown in the figure; the sequence of the reverse primer is complementary to that shown in the figure.

The increase in G12 strains also requires the development of a rapid detection method by PCR using specific primers that specifically target G12 strains. Although 2 earlier papers have described similar primers, neither amplification conditions nor the validation of their specificity for G12 strains have been described. Since we have encountered cross-reactions with rotaviruses carrying other genotypes, i.e, G10, in particular (data not shown), primers were designed and amplification conditions were optimized so that no cross-reactions occurred with prototype rotavirus strains carrying genotypes G1–11 and G13–14. This primer pair should become a valuable asset for the identification of G12 strains found among the nontypeable specimens in many epidemiologic studies since the primer binding sites are well conserved among G12 strains from diverse locations (Figure 2).
